# Knowledge and performance of nursing students of Kermanshah-Iran regarding the standards of nosocomial infections control: a cross-sectional study

**DOI:** 10.1186/s13104-019-4533-4

**Published:** 2019-08-06

**Authors:** Parastoo Majidipour, Amir Aryan, Maryam Janatolmakan, Alireza Khatony

**Affiliations:** 1 Clinical Research Development Center of Imam Reza Hospital, Kermanshah University of Medical Sciences, Kermanshah, Iran; 2 Students Research Committee, Kermanshah University of Medical Sciences, Kermanshah, Iran; 3 Health Institute, Social Development and Health Promotion Research Center, Kermanshah University of Medical Sciences, Kermanshah, Iran; 4 Nursing Department, School of Nursing and Midwifery, Doolat Abaad, Kermanshah, Iran

**Keywords:** Knowledge, Nosocomial infection, Nursing students, Performance, Iran

## Abstract

**Objectives:**

The aim of this study was to determine the level of knowledge and performance of nursing students in regard to the standards of nosocomial infections control.

**Results:**

The average score of knowledge and performance of the subjects was 12.49 ± 2.3 from 18 and 43.07 ± 0.67 from 54, respectively. There was a direct and significant correlation between the knowledge and performance of students (r = 0.46, *p *< 0.0001). There was no statistically significant relationship between the knowledge of students and variables such as gender and academic year of the students, but there was a significant relationship between students’ performance, and gender (*p *= 0.014) and academic year (*p *= 0.015).

## Introduction

The prevalence of nosocomial infections in developed countries is 7% and in developing countries is 10% [1]. Knowledge and performance of nursing students, as future nurses, play a key role in controlling nosocomial infections [2, 3].

Today, special attention has been paid to the training of nursing students in order to increase their knowledge and performance in preventing and controlling nosocomial infections [4, 5]. Considering the important role of nursing students in managing and controlling nosocomial infections, their knowledge and performance in infection control has been studied in different countries. In a study in Iran, knowledge of 177 nursing students and 162 nursing staff about controlling nosocomial infections was investigated and the results showed that most of nursing staff and students had moderate knowledge [6]. The results of a study in India showed that 75% of the nursing students had good knowledge about the management of nosocomial infections, but their performance was poor [7]. In a study conducted in Jordan (2013), 49.6% of the nursing students had inadequate knowledge about the standards of infection control and 75.9% of them had moderate performance [8]. In another study conducted on nursing students in Jordan, the average knowledge score of the samples was 13.8 out of 18, and about 80% of knowledge-raising questions were correctly answered. Also, the average student performance score was 67.4 out of 80 [9].

The results of these studies indicate a difference in knowledge and performance of nursing students in different countries. Considering the importance of controlling the infection in health care centers, and the lack of knowledge about the level of knowledge and performance of nursing students of Kermanshah-Iran in this regard, the present study was designed and implemented. The purpose of this study was to determine the level of knowledge and performance of 3rd and 4th year nursing students regarding the standards of nosocomial infections control.

## Main text

### Methods

#### Study questions

1. What is the level of knowledge and performance of nursing students about the standards of nosocomial infections control?
2. What is the relationship between the knowledge and performance of nursing students about the standards of nosocomial infections control?
3. What is the relationship between the level of knowledge and performance of nursing students about the standards of nosocomial infections control and their individual characteristics?

#### Sample and sampling method

The study population in this cross-sectional study consisted of all nursing students studying at the Faculty of Nursing and Midwifery of Kermanshah University of Medical Sciences. Samples included all nursing students in the 3rd and 4th year of study (102 students) who were recruited by census method. The inclusion criteria included being at the 3rd and 4th year of BSc nursing program and willing to participate in the study.

#### Instrument

The data collection tool was a researcher-made questionnaire containing three parts. The first part was devoted to demographic information, including 4 questions about age, gender, marital status, and academic year. The second part contained 18 questions and was designed to measure students’ knowledge about the standards of nosocomial infections control. The third part, with 54 questions, was related to the performance of nursing students regarding the standards of Nosocomial infections control. The second and third parts of the questionnaire were developed according to the topics of infection control modules, and previous articles [4, 9, 10]. Validity of the questionnaire was assessed by content validity method, which included two qualitative and quantitative sections. In the qualitative section, a questionnaire was provided to a panel of expert, containing 12 experienced individuals. They were asked to express their views on the questionnaire, and based on their comments; necessary changes were made to the questions. In the quantitative section, according to experts’ opinions, two indexes of the Content Validity Ratio and Content Validity Index were calculated to be 0.78 and 0.81, respectively, indicating the acceptable content validity of the tool. The reliability of the questionnaire’s knowledge section was investigated by split-half method (r = 0.79). Cronbach’s alpha method was used to determine the reliability of the performance section and the alpha value was determined to be 0.78. Questions of the knowledge section were in the form of yes, no, and I do not know. Score of 1 was given to the correct answers and the wrong answers and “I do not know” received the score of zero. The total number of correct answers was considered to be the knowledge level of the samples. The score range of the knowledge questionnaire was 0–18.

The third part of the questionnaire was devoted to the performance of the students and included 54 questions with the “I do” and “I do not do” response, and students had to choose one of them that was closer to their performance. This questionnaire had five sections, including suctioning (10 questions), intravenous injection (7 questions), cannula insertion (15 questions), hand-washing (7 questions) and dressing (15 v), which were completed by self-report. Each one of the “I do” and “I do not do” options received the score of 1 and 0, respectively. The total score of the questionnaire was between 0 and 54.

#### Data collection

To collect data, first the approval was obtained from the Ethic Committee of University. The researcher then attended the Education Department of school of nursing and midwifery and obtained the education schedule of the 3rd and 4th year students. Then, according to the schedule, students were selected. For this purpose, the study objectives were first explained to the samples and their consent to participate in the study was obtained. Then, the questionnaires were given to the samples and after compilation were collected by the researcher.

#### Data analysis

Data were analyzed using 18th version of the Statistical Package for Social Sciences. First, the Kolmogorov–Smirnov test was performed, which indicated an abnormal distribution of performance and knowledge scores of the samples. To determine the relationship between the knowledge and performance scores of the students and their gender and academic year, the U Mann–Whitney test was used. To determine the relationship between knowledge and performance, Spearman’s correlation coefficient was used. The significance level for all tests was less than 0.05.

#### Ethical considerations

The Ethical Review Committee of the University approved the study with reference number: IR.KUMS.REC.1398.378. A written informed consent was obtained from all participants and emphasis was placed on the confidentiality of personal information.

### Results

In our study 48% of the students (n = 49) were female and 67.6% (n = 69) were single. The mean age of the subjects was 23.83 ± 1.81 years. Half of the subjects were in the 3rd year and the other half were in the 4th year of nursing education (Table 1).

**Table 1:** Demographic characteristics of students (n = 102)

Variables	n (%)
Gender	
Female	49 (48)
Male	53 (52)
Educational year	
3rd	51 (50)
4th	51 (50)
Marital status	
Married	52 (32.4)
Single	69 (67.6)

The average students’ knowledge about the standards of nosocomial infections control was 12.49 ± 2.3 from 18 (CI 95%, 11.83–12.89). The mean of knowledge score of male and female students were 13.00 ± 2.6 and 11.88 ± 1.8, respectively. This difference was not statistically significant. The average score of knowledge of students in the 3rd and 4th year of study was 12.82 ± 1.9 and 12.14 ± 2.7, respectively which showed no statistically significant difference (Table 2).

**Table 2:** Mean of knowledge and performance of nursing students in terms of demographic variables

Variables	Knowledge		Performance	
	Mean ± SD	*p*-value	Mean ± SD	*p*-value
Gender				
Female	12.80 ± 2.85	0.131	44.16 ± 5.2	0.014
Male	11.85 ± 2.04		41.78 ± 6.6	
Educational year				
3rd	12.7 ± 2.1	0.173	44.6 ± 3.8	0.015
4th	12.02 ± 2.9		41.6 ± 7.2	
Average of previous semester				
≤ 16	12.15 ± 2.4	2.57	42.43 ± 6.3	0.024
≥ 16.01	13.23 ± 3.03		45.64 ± 3.3	

*SD* standard deviation

The average score of students’ performance was 43.07 ± 0.67 out of 54 (CI 95%, 41.83–44.32). The average performance of female students was significantly higher than male students (*p *= 0.014), (44.16 ± 5.1 and 41.78 ± 6.6, respectively). The average performance score of the 3rd year students was higher than 4th year students (44.6 ± 3.8 and 41.45 ± 7.3 respectively). This difference was statistically significant (*p *= 0.015). The average performance score of students with an average grade of more than 16.1 was significantly better than those with average grade of less than 16 (*p *= 0.024) (Table 2). The results indicated a direct and significant correlation between knowledge and performance of the samples about the standards of nosocomial infections control (r = 0.46, *p *< 0.001).

### Discussion

This study aimed to determine the level of knowledge and performance of nursing students regarding the standards of Nosocomial infections control. We found that the knowledge of nursing students was higher than the average score (12.49 from 18), which did not seem to be sufficient due to the importance of observing the nosocomial infection control standards in the hospital. The results of a number of studies indicate a moderate level of knowledge among nurses [8, 11–13] which are consistent with our results. The results of some studies also show that nurses are well aware of the standards of infection control [14, 15]. Evidence suggests that inadequate knowledge of infection control standards creates fear and reluctance to care for the patient in students, which is harmful to their performance [16]. It seems that revising the nursing curriculum and raising the ceilings of units related to infection control measures would be a useful measure to increase the knowledge and enhance the performance of nursing students. In this regard, the use of web-based methods can provide students with educational content. Evidence also suggests the effectiveness of these methods in increasing the knowledge of nurses [17].

In our study, the average performance of nursing students about the standards of nosocomial infections control was relatively adequate. However, it should be at desirable level considering the risks that nosocomial infections pose to patients. The results of some studies indicate the moderate level of nurses’ performance in standards of infection control [11, 13], which is consistent with the results of our study. The results of a study showed that poor nurses’ performance in a hospital in Zambia was related to the standards of infection control [14]. The results of some studies also indicated that nurses’ performance in infection control measures was at adequate level [12, 15]. Considering the importance of using infection control standards in the patient’s bedside, more emphasis should be placed on the control of nosocomial infections in theoretical and clinical modules.

We found a significant correlation between the knowledge and performance of nursing students about the standards of nosocomial infections control. However, this finding was not unexpected, because increased knowledge should lead to improved performance. Research evidences suggest that performance improves as knowledge increases [18–20]. However, in some studies, no significant relationship has been found between the knowledge and performance of nurses about infection control measures [9, 11], which is not consistent with our findings.

In our study, female students had better knowledge and performance about the standards of nosocomial infections control compared to male students. However, this difference was significant only in terms of performance. In some studies a significant relationship has been reported between gender and performance [11, 21, 22]. In a number of studies, a significant relationship has also been found between the knowledge of nurses and their gender [13, 21, 23]. However, no relationship was found between gender and knowledge of nurses in another study [11]. We believe that all nursing students, both male and female, should be competent in theoretical knowledge and clinical practice in terms of infection control measures.

In our study, the knowledge of 3rd and 4th year students was higher than the average, and there was no significant difference between them. Our findings are in line with the results of Qasmi and Ebrahimpour studies [24, 25]. In our view, all students entering the clinical setting must have sufficient knowledge about the infection control.

In our study, the average performance score of 3rd year students in infection control was significantly higher than 4th year students. Although the performance score of 3rd-year students was higher, it was far from the ideal situation. Infection control is one of the responsibilities of nurses and nursing students at the clinical setting, and it is imperative for patient care providers to have sufficient knowledge and skills in this regard to prevent patients becoming infected.

## Limitations

We did not assess the attitude of students, while attitude can have a profound effect on students’ knowledge and performance. Therefore, it is recommended that attitudes of participants should be taken into consideration in future research. Due to the nature of cross-sectional studies, it was not possible to determine the cause and effect relationship between the variables.

## Data Availability

The datasets used during the current study are available from the corresponding author on reasonable request.
